# Affordability of Heathy, Equitable and More Sustainable Diets in Low-Income Households in Brisbane before and during the COVID-19 Pandemic

**DOI:** 10.3390/nu13124386

**Published:** 2021-12-08

**Authors:** Amanda J. Lee, Dori Patay, Lisa-Maree Herron, Ru Chyi Tan, Evelyn Nicoll, Bronwyn Fredericks, Meron Lewis

**Affiliations:** School of Public Health, Faculty of Medicine, The University of Queensland, 266 Herston Rd, St Lucia, QLD 4006, Australia; d.patay@uq.edu.au (D.P.); l.herron@uq.edu.au (L.-M.H.); ruchyi.tan@uq.edu.au (R.C.T.); e.nicoll@uq.net.au (E.N.); b.fredericks@uq.edu.au (B.F.); m.lewis@uq.edu.au (M.L.)

**Keywords:** food prices, food security, diet prices, diet affordability, COVID-19, healthy diet

## Abstract

The COVID-19 pandemic has increased food insecurity worldwide, yet there has been limited assessment of shifts in the cost and affordability of healthy, equitable and sustainable diets. This study explores the impact of the COVID-19 pandemic and income supplements provided by the Australian government on diet cost and affordability for low-income households in an Australian urban area. The Healthy Diets ASAP method protocol was applied to assess the cost and cost differential of current and recommended diets before (in 2019) and during the COVID-19 pandemic (late 2020) for households with a minimum-wage and welfare-only disposable household income, by area of socioeconomic disadvantage, in Greater Brisbane, Queensland, Australia. Data were collected between August and October, 2020, from 78 food outlets and compared with data collected in the same locations between May and October, 2019, in an earlier study. The price of most healthy food groups increased significantly during the pandemic—with the exception of vegetables and legumes, which decreased. Conversely, the price of discretionary foods and drinks did not increase during the pandemic. The cost of the current and recommended diets significantly increased throughout this period, but the latter continued to be less expensive than the former. Due to income supplements provided between May and September 2020, the affordability of the recommended diet improved greatly, by 27% and 42%, for households with minimum-wage and welfare-only disposable household income, respectively. This improvement in the affordability of the recommended diet highlights the need to permanently increase welfare support for low-income families to ensure food security.

## 1. Introduction

Access to a healthy diet is a basic human right [1]. Yet poor diet is a major contributor to the global burden of disease [2] and health inequities [3] and also impacts environmental sustainability [4]. The COVID-19 pandemic has increased the risk of household food insecurity worldwide [5] and in Australia [6]. However, there has been limited assessment of shifts in the cost and affordability of current (unhealthy) and recommended (healthy, equitable and more sustainable) diets due to the COVID-19 pandemic.

Less than one percent of Australians consume a diet consistent with recommendations in national guidelines [7]. While recent evidence indicates that recommended diets would be less expensive than current diets in Australia [8,9,10,11,12,13,14], recommended diets would not be affordable for low-income households [9,10].

To ameliorate the expected economic downturn due to the COVID-19 pandemic, in May 2020, the Australian Government provided a one-off cash payment and fortnightly supplements to recipients of a certain range of welfare payments and eased eligibility requirements for some welfare benefits (Economic Support Payment and Coronavirus Supplements, ESPCS) [15]. This study aims to assess the impact of the ESPCS provided between May and September 2020 on the cost and affordability of current and recommended diets in low-income households in the urban capital city of Brisbane and surrounding area (Greater Brisbane) in Queensland, Australia.

## 2. Materials and Methods

The Healthy Diets ASAP (Australian Standardised Affordability and Pricing) method protocol [11] was applied to assess the cost, cost differential and affordability of current and recommended diets before (from May to October 2019) and during (from August to October 2020) the COVID-19 pandemic in households with minimum wage and welfare-only incomes, by area of socioeconomic disadvantage, in Greater Brisbane, the capital city of the state of Queensland, in Australia. Household incomes and diet affordability were calculated according to the protocol, both in 2019 and 2020, with the latter incomes including the ESPCS. The Healthy Diets ASAP method protocol follows the optimal approach of the International Network for Food and Obesity/NCD Research, Monitoring and Action Support (INFORMAS) [16] and resolves the limitations of earlier efforts to measure diet cost and affordability in Australia [17,18]. The protocol comprises five sections: standardised current (unhealthy) and recommended (healthy, equitable and more sustainable) diet pricing tools; store location and sampling; calculation of household incomes; food price data collection; and analysis and reporting [11]. The protocol has been described in detail elsewhere [11] and applied in multiple studies [8,9,10,11,12,13,14].

### 2.1. Diet Pricing Tools

The diet pricing tools include the types and quantities of the foods and drinks in the current and recommended diets for a reference household of four (an adult male and female of 31–50 years of age, a 14-year-old boy and an 8-year-old girl) per fortnight [11]. The current diet is based on the most recently available reported national dietary intake data from the Australian Health Survey National Nutrition and Physical Activity Survey (NNPAS) 2011–2013 [19]. It includes healthy foods and drinks, but in lower amounts than those recommended by the Australian Dietary Guidelines (ADGs) and also discretionary foods and drinks—that is, those defined by the ADGs as not being necessary for health and high in saturated fat, added sugar, sodium and/or alcohol [7]. The recommended diet contains only healthy foods and drinks in the quantities prescribed by the ADGs [7]. Per reference household, the current diet provides 33,869 kJ/day and the recommended diet provides 33,610 kJ/day [7]. The recommended diet is not only healthier and more equitable, but more sustainable; its production uses less water, better supports biodiversity [7] and releases 25% lower greenhouse gas emissions than is the case for the current diet [20]. The detailed list of components of both diets is provided in Supplementary File S1.

### 2.2. Store Location and Sampling

Baseline price data for 2019 were sourced from an earlier study (the 2019 survey) in which the costs and affordability of the current and recommended diets were assessed in Queensland according to the Healthy Diets ASAP protocol [11]. In the 2019 survey, Statistical Area Level 2 (SA2) locations throughout the state (the Australian Bureau of Statistics classes medium-sized geographical areas into SA2 locations, where communities “interact together socially and economically” [19]) were stratified into quintiles of socioeconomic disadvantage based on the Index of Relative Socioeconomic Disadvantage (IRSD) [21]. In total, 18 SA2 locations were randomly selected for inclusion, including 10 in Greater Brisbane. The most disadvantaged SA2 areas were in SEFIA quintile 1, the median disadvantaged SA2 areas were in SEIFA quintile 3 and the least disadvantaged SA2 areas were in SEIFA quintile 5.

In 2020, because of public health measures including travel restrictions that were implemented due to the COVID-19 pandemic, food prices could not be collected outside Greater Brisbane. Therefore, only the 10 SA2 locations (and specific food outlets) surveyed in Greater Brisbane in the 2019 study [9] were included for comparison. At each location, two large supermarkets (one of each major supermarket chain), an independent grocer, a bakery, a fish and chip shop, two fast-food restaurants and one alcohol store were surveyed. One supermarket chain did not wish to have additional personnel visiting their stores during the pandemic, so food and drink prices in these outlets were collected from the chain’s online store in the relevant location. Previous studies comparing in-store to online prices have found insignificant price differences [22].

### 2.3. Calculation of Household Incomes

Disposable household incomes for those on minimum wage and those receiving welfare only were calculated for a reference household according to the Healthy Diets ASAP protocol for low disposable household income [11]. Calculations were based on a set of assumptions detailed in the protocol (included in Supplementary File S2) and informed by revised government payment data from Services Australia [23].

The calculations of the minimum wage and welfare-only disposable household incomes for 2020 included the ESPCS provided between May and September 2020 [15]. This included a one-off AUD 750 payment (Economic Support Payment for eligible low-income households) and a fortnightly AUD 550 payment for those individuals eligible for full or part payment of unemployment benefits (JobSeeker). For inclusion in the household fortnightly income calculations, the AUD 750 payment was divided by the number of fortnights (six) in the study period (Supplementary File S2). Changes to eligibility criteria in 2020 for unemployment benefits meant household members also receiving very low incomes from employment became eligible for both part payment of JobSeeker and the Coronavirus Supplement [15]. During the same period, the government provided an additional payment (JobKeeper) for qualifying (not all) businesses adversely affected by the public health measures introduced during the COVID-19 pandemic, to help cover the costs of employees’ wages (AUD 1500 per fortnight; reduced to AUD 1200 (Tier 1) or AUD 750 (Tier 2) per fortnight from September 2020), so that more employees could continue to earn an income and retain connection with their employers [15]. This supplement was not included in the minimum wage disposable income calculations, as the funds were paid directly to employers rather than employees and it was difficult to determine how many minimum wage earners received such support and any quantum they received.

### 2.4. Price Data Collection

Price data were collected from August to October 2020, by four trained research assistants (E.N., R.C.T., L.-M.H. and D.P.) strictly following the Healthy Diets ASAP protocol [11], using an online platform developed for this purpose or paper-based survey forms (Supplementary File S3). Before data collection commenced, permission was requested and received from national head offices of large supermarket chains and also from store managers at each food outlet.

### 2.5. Analysis and Reporting

The food price data were double entered in the Healthy Diets ASAP data collection website and cleaned by EN and RCT; the website generated reports in Microsoft^®^ Office Excel (2016) files for each location. The data in these reports were cross-checked by DP and ML.

The mean cost of the current and recommended diets and cost of each food group in the diets were calculated for the reference household per fortnight for each SA2 location. Diet affordability was calculated for households with minimum wage and welfare-only disposable household incomes. Diet costs and affordability were then calculated by area of socioeconomic disadvantage (for SEIFA quintiles 1, 3 and 5). In Australia, a diet is deemed to be unaffordable if it costs 30% or more of household income; if the cost is between 25% and 30% of household income, the household is considered to be under food stress [24]. To assess change in diet costs, cost of component food groups and affordability of the diets before and during the COVID-19 pandemic, the results were compared to the relevant findings of the 2019 survey. The statistical significance of any differences between the 2019 and 2020 results and by area of socioeconomic disadvantage were assessed by a *t*-test after the analysis of variance confirmed that the data were parametric.

## 3. Results

### 3.1. Selected Locations and Stores

In the Greater Brisbane region surveyed, three SA2 locations were in areas classified as SEIFA quintile 1, four in quintile 3 and three in quintile 5. Three food outlets—an independent supermarket, a bakery and a fish and chip store—had closed since 2019. A similar independent grocery store in the SA2 area was surveyed. In the other two locations, there was no similar food outlet; therefore, mean food prices from the same type of outlets in other locations with the same SEIFA quintile were used. In 2019, 80 food outlets in Greater Brisbane were included; in 2020, 78 stores were surveyed, with price data collected in-store in 68 food outlets and online for 10 supermarkets.

### 3.2. Cost of Current and Recommended Diets

**Figure 1:**
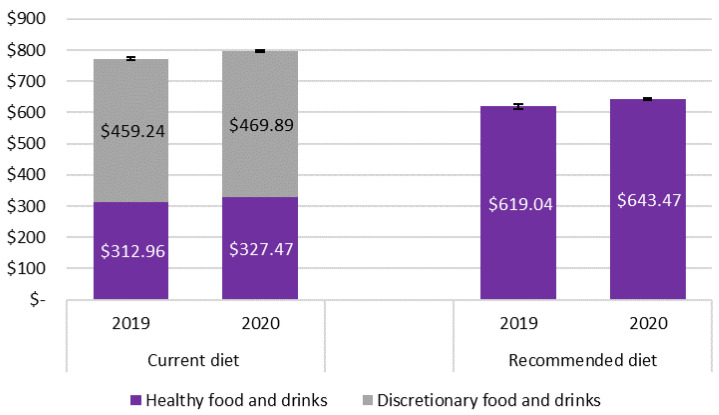
Cost of the current diet (and discretionary and healthy components) and the recommended diet in 2019 and 2020 in Greater Brisbane, for a reference household per fortnight (AUD). (Error bars indicate the standard error).

Table 1 presents the mean cost of current and recommended diets, cost of diet components (by ADG food group and as either discretionary or healthy food categories) and change in costs in Greater Brisbane from 2019 to 2020. Figure 1 summarises Table 1, depicting total diet costs and the costs of the healthy and discretionary components of the diets. Detailed diet cost data for each SA2 location and by area of socioeconomic disadvantage are presented in Supplementary Files S4 and S5, respectively. Differences between the mean costs of the total diet in 2019 and 2020 for each of the SEIFA quintiles and between quintiles, are provided in Supplementary File S6.

In Greater Brisbane, both in 2019 and 2020, the current diet (AUD 772.20 ± 14.18 and AUD 797.36 ± 12.00 per fortnight, respectively) was significantly more expensive than the recommended diet (AUD 619.04 ± 22.66 and AUD 643.47 ± 18.48 per fortnight, respectively (*p* < 0.01)) (Table 1, Figure 1). For the reference household, per fortnight, the recommended diet would have been 20% (AUD 153.16) less expensive than the current diet in 2019 and 19% (AUD 153.89) less in 2020. The recommended diet was less expensive than the current diet in each SA2 area surveyed (Supplementary File S4). In Greater Brisbane, there was no significant difference between the costs of the diets in different areas of socioeconomic disadvantage (Supplementary File S6).

Between 2019 and 2020, the cost of both the current (3.3%, *p* = 0.01) and recommended (4.0%, *p* < 0.5) diets increased significantly in Greater Brisbane (Table 1); the mean cost of the recommended diet increased 21% more than the current diet. In the current diet, the prices of healthy foods increased significantly, by an average of 4.6% (*p* < 0.01), but the prices of discretionary foods and drinks did not change significantly over the year. In the recommended diet, the food groups that increased the most in cost were fruits (22.3%, *p* < 0.01); milk, yoghurt, cheese and alternatives (7.4%, *p* < 0.5); and lean meat, poultry, fish, eggs and alternatives (6.1%, *p* < 0.01) (Table 1). ‘Vegetables and legumes’ was the only food group that decreased in cost over the year (−12.1%, *p* < 0.01) (Table 1).

In both years, discretionary food and drinks cost nearly 60% of the reference household’s expenditure on diet (Table 1). The items comprising the highest proportion of expenditure on discretionary food and drinks were takeaway foods (19–20%) and alcoholic drinks (12–13%) (Table 1).

### 3.3. Affordability of Current and Recommended Diets

The minimum-wage disposable household income in Greater Brisbane was AUD 2358.33 per fortnight in August 2019; this increased to AUD 3336.20 between May and September 2020 due to the ESPCS [15]. The welfare-only disposable household income was AUD 1739.68 per fortnight in August 2019; this increased to AUD 3082.52 per fortnight with the additional ESPCS in 2020 [15].

**Figure 2:**
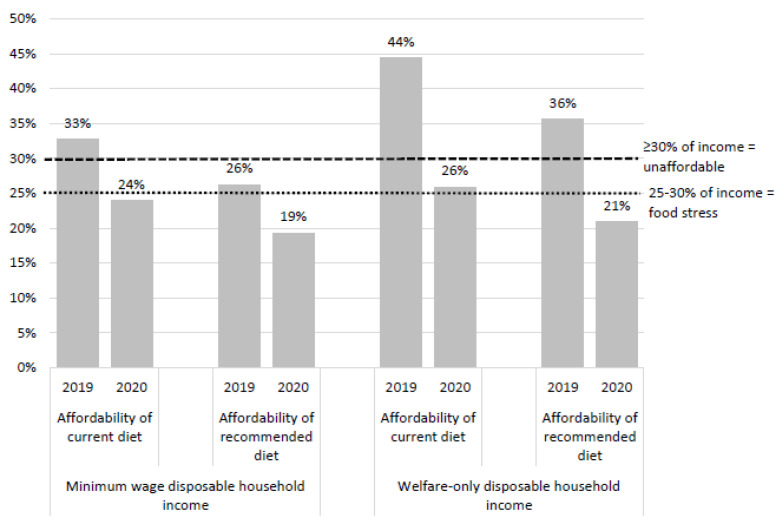
Affordability of current and healthy diets for a reference household with low disposable income in Greater Brisbane.

Affordability of current and recommended diets for the reference household with minimum-wage and welfare-only disposable household income in 2019 and 2020 is presented in Figure 2. Detailed diet affordability data by area of socioeconomic disadvantage are provided in Supplementary File S7.

The recommended diet would have cost 26% of the disposable income of minimum wage households in 2019, placing households under food stress. However, with the ESPCS, the recommended diet would have been affordable for these households, costing only 19% of disposable income in 2020 (Figure 2). In 2019, the recommended diet was unaffordable for welfare-only households as it cost 36% of disposable income; in 2020, with the ESPCS, this proportion dropped to 21%, making recommended diets affordable for the first time in these vulnerable households (Figure 2). Affordability of recommended diets was consistent across areas of socioeconomic advantage in Greater Brisbane (Supplementary File S7). Due to the ESPCS, from 2019 to 2020, the affordability of recommended diets improved by 27% for households on minimum wage and by 42% for households on welfare-only income (Figure 2).

## 4. Discussion

Many factors affect food choice. These include taste, healthiness/nutrition, convenience and cultural acceptability; availability and accessibility of different foods; promotions and marketing; and the perception that healthy food and drinks cost more than discretionary items [7]. In addition, food and alcohol industry marketing (e.g., advertising and promotions) and other practices (such as government lobbying) can help drive consumer choice of discretionary foods and drinks [25], which account for the majority of household expenditure on diet in Australia [12,19]. The influence of such factors might explain why households in Greater Brisbane continue to purchase the current diet despite the recommended diet being 19% less expensive. This corresponds to findings of prior research using the Healthy Diets ASAP protocol in Australia that has shown that, under current policy settings, which include the exemption of “basic, healthy foods” from the Goods and Services Tax (GST) [9,10], the current diet is from 14% to 23% more expensive than the recommended diet in Brisbane [11,12], Canberra and Sydney [10], regional Victoria [13], Aboriginal communities in remote Australia [8] and in two large supermarket chains nationally [14].

Our results that the recommended diet can be less expensive than the current diet also correspond to the findings of studies using methods other than Healthy Diets ASAP in the Northern Territory of Australia [26], throughout Australia [27], in New Zealand [28] and in Mexico [29]. While a systematic review [30] and two relevant studies [31,32] reported contradicting results, methodological differences in the contents of the items priced (for example, variation in inclusion/exclusion of alcoholic beverages and ‘take-away’ foods) and units of cost results (for example, by energy, weight, serve or nutrient density) render a comparison problematic [17]. This highlights the importance of ‘anchoring’ foods and drinks to be priced in total diets and using standardised methods to support cost comparison [16,17,18]. Commonly, it is presumed that nutritious foods are more expensive than unhealthy foods and that this is a major driver of dietary inequities, particularly during the COVID-19 pandemic [3]; therefore, our study illustrates the need for more granular country-specific and regional studies into food and diet affordability over time.

Findings that the current diet cost increased by 3.3% correspond to the Consumer Price Index of food and non-alcoholic beverages of 3.4% between 2019 and 2020 [33]. This increase is likely not only due to the COVID-19 pandemic, but also the extended drought and the unprecedented bushfires in Australia in 2019 and early 2020 [33], as well as the effect of an outbreak of African swine fever in China in 2019 that impacted meat prices in Australia [34]. This study found that the price of healthy foods and drinks increased more than discretionary choices over the year. Most healthy ADGs food groups (including fruit; milk, yoghurt, cheese and alternatives; and lean meats, poultry, fish, eggs and plant-based alternatives) increased in price. However, the price of vegetables and legumes decreased over the year. No comparable granular data exploring price changes in specific food groups were identified in other studies in Australia during this time. The increasing relative price of healthy foods compared to discretionary foods during the COVID-19 pandemic may help explain the reduced intake of fresh produce identified in several studies in 2020 [35] and be a factor for the increased intake of discretionary ‘comfort’ or ‘snack’ foods and drinks and weight gain reported by many during this period [35,36,37].

Despite the increased price of most healthy foods between 2019 and 2020, this study demonstrates that the affordability of recommended diets improved dramatically for low-income families receiving the ESPCS, with many being lifted out of poverty. A survey by the Australian Council of Social Science (ACOSS) found that 93% of interviewed ESPCS recipients reported that they could afford more healthy foods, including fruits and vegetables, than before the supplement was provided [38]. Increased income support was likely essential to help low-income households afford sufficient food, as reduced incomes due to impacts of COVID-19 restricted their capacity to meet essential expenses. In mid-2020, about 25% of Australian renters in the private rental market reported skipping meals to save money [39]. Around 40 per cent of people surveyed indicated that after paying rent, there was not enough money left to pay for essentials including bills, transport and food [40]. More recent data during the pandemic show that, of the extra income received due to the coronavirus supplements, 15% was spent on household bills (electricity, phone and water), 13% on food, 11% on clothing and household goods and around 32% saved or used to pay down debt [41]. Although nutrition education is an important component of food system transformation [3], these data reinforce previous findings that it is not just lack of education, but rather lack of supportive regulatory policy action that is the key barrier to diet equity in developed economies [42].

Few studies assessing the impact of changing affordability of recommended diets due to the impacts of the COVID-19 pandemic have been published to date, but available data show a positive impact of income support on food security and ability to buy healthy food. One challenge in interpreting global comparisons is that different definitions of diet affordability are used in different regions of the world; for example, in Europe and Central Asia, ‘affordability’ is set at 63% of the poverty line [43]. In high-income countries most comparable to Australia, among those who lost their job due to the COVID-19 pandemic in the United States of America (USA), provision of unemployment insurance reduced food insecurity by 35% in low-income households [44]. In addition, in the USA, the proportion of households with children reporting insufficient food declined in the month following the expansion of child tax credit payments [45]. In the United Kingdom (UK), the prevalence of food-related hardships (reported inability to eat healthy or nutritious food or being hungry but not eating) escalated between April and July 2020, with the largest increases among those who had lost employment; studies found that the UK government income supplements (the Coronavirus Job Retention Scheme and Self-Employment Income Support Scheme) “appeared to have conferred some protection” [46].

However, in Australia, by the end of 2020, the Coronavirus Supplement had been gradually decreased to AUD 150 per fortnight and ceased from March 2021, with the government lifting the ongoing base rate of the JobSeeker payment from pre-COVID levels by AUD 50 a fortnight from 1 April 2021. This means that most people on JobSeeker earn about AUD 620 a fortnight, or AUD 44 a day [47]. Survey results show food insecurity has increased since the supplement was withdrawn, with one in six respondents being categorised as “severely food insecure” and nearly a third of people having been reported to be struggling to meet their food needs, experiencing food insecurity for the first time [48]. Almost half (48%) of the respondents who had accessed JobSeeker (and JobKeeper) payments reported not to be coping well since the additional support has ended [48]. This underlines the importance of permanently increasing income support for those on welfare in Australia, ideally at least to the levels that were provided between May and September 2020 [49]. The Australian Council of Social Services (ACOSS) has proposed that JobSeeker should be increased to at least AUD 65 per day, which would raise it to just above the poverty line [47].

As previously noted [12], the findings also suggest that more needs to be done to improve the relative affordability of recommended diets compared to current diets. This includes protecting the exemption of basic, healthy foods from GST in Australia [9,12]. However, expanding the base of the GST to include basic, healthy foods is mooted regularly in Australia [12]. Conversely, previous studies and modelling suggest that the 10% tax differential should be increased to 20% to help drive preference for healthy foods and drinks [12,50]. Recently, the Organisation for Economic Co-operation and Development has called on Australia to raise the GST and to also increase unemployment benefits [51].

## 5. Limitations

The Healthy Diets ASAP protocol has inherent methodological limitations, discussed elsewhere [11]. For example, the disposable income calculation for the minimum-wage and welfare-only household represents only two particular situations and sets of assumptions. It does not include JobKeeper in the minimum-wage disposable income calculations, although some low-income households were likely to receive this supplement between May and September 2020. Therefore, this study may not identify affordability challenges facing some families who have experienced job losses and/or reduced income due to the pandemic.

Recent granular data on current diet patterns were not available, as national level dietary intake data have not been collected in Australia since 2011-2012 [52,53]. The changing food environment in recent years, such as the rise of online meal delivery services and increased use of these in lockdowns during the COVID-19 pandemic [54,55], are likely to have changed the usual dietary intake of the population.

Finally, it is essential to acknowledge that the COVID-19 pandemic impacted food environments in remote and rural areas differently from capital cities, with reports of rural and remote communities experiencing greater disruptions to food supply [56,57], especially in remote Aboriginal and Torres Strait Islander communities [58]. Hence, the impacts on diet costs in rural and remote areas would likely be different from the results of this study in Greater Brisbane. Further, as outlined clearly in a recent report on the impacts of COVID-19 on Indigenous communities throughout Australia [58], some populations responded resourcefully to the crisis to help protect themselves and safeguard their own food security. The degree to which the observed changes in food costs may be generalisable to other locations in Australia, or other countries internationally, is currently unknown.

## 6. Conclusions

Before the COVID-19 pandemic, in Greater Brisbane, recommended diets were unaffordable for welfare dependent families and households living on minimum wage were subject to food stress. This study found that the costs of both current and recommended diets increased significantly between 2019 and 2020 during the COVID-19 pandemic. The price of most healthy food groups increased significantly during the pandemic—with the exception of vegetables and legumes, which decreased. Conversely, the price of discretionary foods and drinks did not increase during the pandemic. While the cost of the recommended diet increased more than the current diet, it continued to be less expensive than the latter during this period.

Despite these rising costs, the ESPCS provided by the Australian Government improved affordability of recommended diets and, for the first time, welfare dependent families had economic access to recommended diets. A permanent increase of welfare income for low-income households would improve food security and diet-related health, which is particularly critical in the midst of a global pandemic. In the absence of such ongoing support, now that the supplements have been reduced, it will be imperative to continue to monitor the cost and affordability of heathy, equitable and more sustainable diets—both in low-income households in Brisbane and, where public health restrictions allow, in other locations, including remote and Aboriginal and Torres Strait Islander communities—during and after the COVID-19 pandemic to inform essential health and economic policy action.

## Figures and Tables

**Table 1:** Mean costs of diet and components and change from 2019 to 2020 in Greater Brisbane.

	Total Diet and Food Group Costs of the Current Diet for a Reference Household Per Fortnight						Total Diet and Food Group Costs of the Recommended Diet for a Reference Household Per Fortnight					
	2019		2020		Change in Cost from 2019 to 2020 (AUD)	Change in Cost from 2019 to 2020 (%)	2019		2020		Change in Cost from 2019 to 2020 (AUD)	Change in Cost (%)
Food/Food Groups	Mean Cost ± SD (AUD) (n = 30 Locations)	Proportion of Total Diet Cost (%)	Mean Cost ± SD (AUD) (n = 30 Locations)	Proportion of Total Diet Cost (%)			Mean Cost ± SD (AUD) (n = 30 locations)	Proportion of Total Diet cost (%)	Mean Cost ± SD (AUD) (n = 30 Locations)	Proportion of Total Diet Cost (%)		
Water, bottled	AUD 20.35 ± 1.64	2.64%	AUD 19.04 ± 1.75	2.39%	−AUD 1.32	−6.47%	AUD 20.35 ± 1.64	3.29%	AUD 19.04 ± 1.75	2.96%	−AUD 1.32	−6.47%
Fruits	AUD 53.38 ± 4.08	6.91%	AUD 57.44 ± 2.26	7.20%	+AUD 4.06 *	+7.60%	AUD 72.81 ± 7.97	11.76%	AUD 88.31 ± 6.16	13.72%	+AUD 15.50 **	+21.29%
Vegetables (and legumes)	AUD 43.59 ± 1.88	5.65%	AUD 40.51 ± 1.69	5.08%	−AUD 3.08 **	−7.07%	AUD 110.36 ± 5.38	17.83%	AUD 96.97 ± 4.52	15.07%	−AUD 13.39 **	−12.13%
Grain (cereal) foods	AUD 44.34 ± 1.98	5.74%	AUD 46.17 ± 1.64	5.79%	+AUD 1.83	+4.12%	AUD 109.99 ± 2.36	17.77%	AUD 113.86 ± 3.95	17.69%	+AUD 3.87	+3.52%
Lean meats, poultry, fish, eggs, nuts, seeds and alternatives	AUD 96.45 ± 3.63	12.49%	AUD 101.86 ± 4.14	12.77%	+AUD 5.41 *	+5.61%	AUD 184.52 ± 8.71	29.81%	AUD 195.83 ± 7.56	30.43%	+AUD 11.31 **	+6.13%
Milk, yoghurt, cheese and alternatives	AUD 47.93 ± 2.75	6.21%	AUD 55.02 ± 1.33	6.90%	+AUD 7.08 **	+14.78%	AUD 112.59 ± 8.35	18.19%	AUD 120.88 ± 5.35	18.79%	+AUD 8.28 *	+7.36%
Unsaturated oils and spreads	AUD 1.27 ± 0.06	0.17%	AUD 1.30 ± 0.06	0.16%	+AUD 0.02	+1.86%	AUD 8.42 ± 0.38	1.36%	AUD 8.59 ± 0.39	1.33%	+AUD 0.17	+1.98%
Artificially sweetened beverages	AUD 5.64 ± 0.43	0.73%	AUD 6.14 ± 0.21	0.77%	+AUD 0.50 **	+8.82%	-	-	-	-	-	-
Sugar sweetened beverages	AUD 31.14 ± 1.57	4.03%	AUD 30.82 ± 1.00	3.87%	−AUD 0.32	−1.04%	-	-	-	-	-	-
Takeaway foods	AUD 149.31 ± 7.01	19.34%	AUD 157.76 ± 7.40	19.79%	+AUD 8.44 **	+5.65%	-	-	-	-	-	-
Alcoholic beverages	AUD 96.36 ± 6.05	12.48%	AUD 97.93 ± 2.87	12.28%	+AUD 1.57	+1.63%	-	-	-	-	-	-
All other discretionary choices	AUD 182.42 ± 9.50	23.62%	AUD 183.38 ± 6.62	23.00%	+AUD 0.96	+0.53%	-	-	-	-	-	-
**Total diet**	**AUD 772.20 ± 14.18**	**100%**	**AUD 797.36 ± 12.00**	**100.00%**	+AUD 25.15 *	**+3.26%**	**AUD 619.04 ± 22.66**	**100%**	**AUD 643.47 ± 18.48**	**100%**	+AUD 24.42 *	**+3.95%**
Healthy foods and drinks in total diet	AUD 312.96 ± 10.21	40.53%	AUD 327.47 ± 8.06	41.07%	+AUD 10.65 **	+4.63%	AUD 619.04 ± 22.66	100%	AUD 643.47 ± 18.48	100%	+AUD 24.42 *	+3.95%
Discretionary foods and drinks in total diet	AUD 459.24 ± 7.54	59.47%	AUD 469.89 ± 9.16	58.93%	+AUD 14.50	+2.32%	-	-	-	-	-	-

* indicates *p* < 0.05; ** indicates *p* < 0.01.

## Data Availability

The data presented in this study are available, in a de-identified format, on request from the corresponding author. The data are currently held in a secure research environment according to ethical requirements.

## Supplementary Materials

The following are available online at https://www.mdpi.com/article/10.3390/nu13124386/s1, Supplementary File S1: Details of the current and recommended diets: total energy of diets and goods comprising the diets, per reference household per fortnight, Supplementary File S2: Calculations of minimum wage and welfare-only disposable household incomes for a reference household per fortnight, Supplementary File S3: The price data collection form, Supplementary File S4: Total diet and component costs for a reference household per fortnight in each included location in Greater Brisbane, Supplementary File S5: Total diet and component costs for a reference household per fortnight in Greater Brisbane by area of socioeconomic disadvantage, Supplementary File S6: Differences between the mean total cost of the current and recommended diets by area of socioeconomic disadvantage and within area of socioeconomic disadvantage, Supplementary File S7: Affordability of current and recommended diets in Greater Brisbane by area of socioeconomic disadvantage.
