# Supplementary material for: Affordability of Heathy, Equitable and More Sustainable Diets in Low-Income Households in Brisbane before and during the COVID-19 Pandemic
Source: Nutrients. 2021 Dec 8;13(12):4386. doi: 10.3390/nu13124386 (PMC8705813; doi:10.3390/nu13124386)
Supplement: Supplementary file 1 [file nutrients-13-04386-s001.zip › nutrients-1471843-supplementary.pdf]

Supplementary File S1. Details of the current and recommended diets: total energy of diets and goods comprising the diets, per reference household per fortnight

Table S1: Total energy of diets and goods comprising the current and recommended diets, per reference household per fortnight

	**Current diet**	**Recommended diet**		**Current diet**	**Recommended diet**		**Current diet**
**Total energy of basket**	**474,040 kJ**	**470,540 kJ**	***Grain (cereal) foods***			***Discretionary choices****	
			Wholegrain cereal biscuits, Weetbix^{TM} (g)	430	2216	Beer, full strength (ml)	4661
			Wholemeal bread, pp (g)	1054	4272	White wine, sparkling (ml)	863
			Rolled oats, whole (g)	870	6648	Whiskey (ml)	266
**Food (per fortnight)**			White bread, pp (g)	3033	893	Red wine (ml)	1078
Bottled water, still (ml)	5296	5296	Cornflakes (g)	680	670	Butter (g)	280
Artificially sweetened beverages (ml)	2391	N/A	White pasta, spaghetti (g)	1326	2042	Muffin, commercial (g)	1455
**Core five food groups**			White rice, medium grain (g)	1622	2042	Cream-filled sweet biscuit, pp (g)	496
Fruit			Dry water cracker biscuit (g)	259	781	Muesli bar, pp (g)	373
Apples, red, loose (g)	3497	5460	Bread in sandwich (g)	120	120	Mixed nuts, salted (g)	255
Bananas, Cavendish, loose (g)	899	5460	***Meats, poultry, fish, eggs, nuts, seeds and alternatives***			Pizza, commercial (g)	1182
Oranges, loose (g)	1664	5460	Beef mince, lean (g)	267	1168	Savoury flavoured biscuits (g)	222
Fruit salad, canned in juice (g)	2046	N/A	Lamb loin chops (g)	257	1169	Confectionary (g)	418
Fruit juice (ml)	3026	N/A	Beef rump steak (g)	1056	1172	Chocolate (g)	441
Vegetables			Tuna, canned in vegetable oil (g)	1052	1841	Sugar sweetened beverages, Coca Cola (ml)	12012
Potato, white, loose (g)	1460	2320	Whole barbecue chicken, cooked (g)	1661	1471	Meat pie, commercial (g)	1638
Sweetcorn, canned, no added salt (g)	206	1160	Eggs (g)	872	2208	Frozen lasagne, pp (g)	4322
Broccoli, loose (g)	422	1470	Meat in tinned meat and vegetable casserole (g)	646	780	Hamburger, commercial (g)	2413
White cabbage, loose (g)	235	1470	Chicken in sandwich (g)	120	120	Beef sausages (g)	1048
Iceberg lettuce, whole (g)	795	1470	Peanuts, roasted, unsalted (g)	N/A	780	Ham (g)	189
Carrot, loose (g)	753	2205	***Milk, yoghurt, cheese and alternatives***			Potato crisps, pp (g)	518
Pumpkin (g)	240	2205	Cheddar cheese, full fat (g)	624	704	Potato chips, hot, commercial (g)	670
Four bean mix, canned (g)	74	1005	Cheddar cheese, reduced fat (g)	44	516	Ice cream (g)	1830
Diced tomatoes, canned in tomato juice (g)	234	1638	Milk, full fat (g)	5961	6438	White sugar (g)	564
Onion, brown, loose (g)	84	1638	Milk, reduced fat (g)	2929	12000	Salad dressing (ml)	277
Tomatoes, loose (g)	488	1638	Yoghurt, full fat, plain (g)	204	2576	Tomato sauce (ml)	569
Frozen mixed vegetables, pp (g)	1184	1638	Yoghurt, reduced fat, flavoured (vanilla) (g)	676	5100	Chicken soup, canned (g)	1340
Frozen peas, pp (g)	273	1638	Flavoured milk (ml)	2416	N/A	Orange juice (ml)	3027
Baked beans, canned (g)	369	1005	***Unsaturated oils and spreads***			Fish fillet crumbed, pp (g)	302
Salad vegetables in sandwich (g)	120	120	Canola margarine (g)	170	412	Instant noodles, wheat based (g)	381
Vegetables in tinned meat and vegetable casserole (g)	646	N/A	Sunflower oil (ml)	7	291		
			Olive oil (ml)	7	291		

** The recommended diet does not contain discretionary choices.*

pp = pre-packaged

Supplementary File S2. Calculations of minimum wage and welfare-only disposable household incomes for a reference household per fortnight

Table S2A Calculations of minimum wage disposable household incomes for a reference household per fortnight

	Reference household: Two parents with two children<br>Adult male (between 31 and 50 years), adult female (between 31 and 50 years), 14 years old boy, 8 years old girl			
**Assumptions**	• The adult male works on a permanent basis at national minimum wage for 38 hours a week ($19.49/hr)<br>• The adult female works on a part-time basis at national minimum wage ($19.49/hr) for 6 hours a week<br>• Both children attend school and are fully immunised<br>• None of the family are disabled<br>• The family has some emergency savings that earn negligible interest<br>• The family is privately renting a 3-bedroom house at $376/week			
	**August 2019**		**May 2020**	
**Income**	**Amount**	**Amount/ fortnight**	**Amount**	**Amount/ fortnight**
Paid employment - adult male	$19.49/hr for 38h/week	$ 1,481.24	$19.49/hr for 38h/week	$1,481.24
Paid employment - adult female	$19.49/hr for 6h/week	$ 233.88	$19.49/hr for 6h/week	$ 233.88
JobSeeker Allowance - adult male	N/A	$ -	N/A	$ -
JobSeeker Allowance - adult female	N/A	$ -	$324.05	$ 324.05
Family Tax Benefit A fortnightly payment	$428.40/fortnight	$ 428.40	$428.40/fortnight	$ 428.40
Family Tax Benefit A annual supplement	$751.90/child/year	$ 57.84	$766.50/child/year	$ 58.96
Family Tax Benefit B fortnightly payment	$107.66/fortnight	$ 107.66	$74.06/fortnight	$ 74.06
Family Tax Benefit B annual supplement	$365.00/year/family	$ 14.04	$372.30/year/family	$ 14.32
Total Clean Energy Supplement (from all payments)	included in Family Tax Benefit estimator amounts	$ -	included in Family Tax Benefit estimator amounts +$7.90	$ 7.90
Rent Assistance	$161.14	$ 161.14	$164.08	$ 164.08
First Economic Support payment	N/A	$ -	$750 paid in April 2020 (assumed spent over 6 fortnights)	$ 125.00
Coronavirus Supplement	N/A	$ -	$550/fortnight from April 27	$ 550.00
Income Tax Paid (fortnightly)	$4063.40/year - low income tax offset ($422.32) - low/med tax offset ($368.42)	$ (125.87)	Tax p.a. less low income tax offset & low-middle income tax offset (nil payable)	$ -
**Fortnightly income total**		**$ 2,358.33**		**$3,336.02**

Source of data: Services Australia (Australian Government website): https://www.servicesaustralia.gov.au/

Table S2B Calculations of welfare-only disposable household incomes for a reference household per fortnight

	**Household 5: Two parents with two children**<br>Adult male, adult female, 14yr boy, 8yr girl			
**Assumptions**	• The adult male is unemployed<br>• The adult female is unemployed<br>• Both children attend school and are fully immunised<br>• None of the family are disabled<br>• The family has some emergency savings that earn negligible interest<br>• The family is privately renting a 3 bedroom house at $376/week			
	**Aug-19**		**May-20**	
**Income**	**Amount**	**Amount/ fortnight**	**Amount**	**Amount/ fortnight**
Paid employment - adult male	N/A		N/A	
Paid employment - adult female	N/A		N/A	
Newstart Allowance - adult male	$504.7/fortnight	$504.70	$510.80/fortnight	$510.80
Newstart Allowance - adult female	$504.7/fortnight	$504.70	$510.80/fortnight	$510.80
Family Tax Benefit A fortnightly payment	$428.40/fortnight	$428.40	$428.40/fortnight	$428.40
Family Tax Benefit A annual supplement	$751.90/child/year	$57.84	$766.50/child/year	$58.96
Family Tax Benefit B fortnightly payment	$52.08/fortnight	$52.08	$31.36/fortnight	$31.36
Family Tax Benefit B annual supplement	$365.00/year/family	$14.04	$372.30/year/family	$14.32
Total Clean Energy Supplement (from all payments)	$7.90 each	$15.80	$7.90 each	$15.80
Rent Assistance	$162.12	$162.12	$164.08	$164.08
Economic Support payment	N/A		2x$750 in 2020	$250.00
Coronavirus Supplement	N/A		2x $550/fortnight from 27 April	$1,100.00
Income Tax Paid (fortnightly)	Nil		Nil	
**Fortnightly income total**		**$1,739.68**		**$3,084.52**

Source of data: Services Australia (Australian Government website): https://www.servicesaustralia.gov.au/

Supplementary File S3. Healthy Diets ASAP (Australian Standardised Affordability and Price) Survey Form

Date: ___________

Store name ____

Store Location: ____

Collector:___________________________

Price Collection Protocol

1. Record the *usual price of an item*, i.e. do not collect the sale/special price unless it is the only price available (if so, note in comment column)
2. *Look for the specified brand and specified size for each food item, and record the price*
   - *If the specified brand is not available*: Choose the cheapest brand (non-generic) available in the specified size. Note this brand in the "Your brand" column
   - *If the specified size is not available*: Choose the nearest larger size in the specified brand. If a larger size is not available, choose the nearest smaller size. Note this size in the "Your size" column.
   - *If both the specified brand and specified size are not available*: Choose the cheapest in the nearest larger size of another brand (non-generic). If a larger size is not available, choose the nearest smaller size.
   - *If multiple brands are specified,* record the price of the cheapest one and note brand in the "Your brand" column
   - *If the item is only available in a generic form* (e.g. Home Brand, Coles, Woolworths Select, Black and Gold) choose the *most expensive generic* item in the specified size. If the specified size is not available, choose the nearest larger size. If a larger size is not available, choose the nearest smaller size. Note the generic name in the "Your brand" and the size in the "Your size" columns.
3. *Loose produce*: choose the usual cheapest price per kg of the variety not on special. If the only variety available is on special, record the special price and note in comments column.
4. *Peanuts*: choose the branded packet size closest to 250g. If packaged, roasted, unsalted peanuts are not available, record the price of the loose 'bulk – scoop & weight' roasted, unsalted peanuts per 100g.
5. *Check all data are recorded as above before leaving store.*

For more information contact Prof Amanda Lee, School of Public Health, The University of Queensland e: Amanda.Lee@uq.edu.au m: 0412975197

Food	Specific brand	Your brand	Specific size	Your size	Your cost	Comments
Fresh Fruit						
Apples, red, loose			per kg			
Bananas, cavendish, loose			per kg			
Orange, loose			per kg			
Fresh Vegetables						
White potato, loose, brushed/washed			per kg			
Broccoli, loose			per kg			
Cabbage, white, ½ cabbage (1/2=1.5kg) (weigh if necessary)			1.5kg			
Lettuce, iceberg, whole (1=0.6kg)			0.6kg			
Carrot, loose			per kg			
Pumpkin, ½ pumpkin (1/2 av. Jap=1.5kg, 1/2 av. Butternut=1kg) (weigh if necessary)			per kg			
Brown onion, loose			per kg			
Tomato, loose (not vine-ripened)			per kg			
Tinned Foods						
Tinned sweet corn, kernels, no added salt	Edgell		420g			
Tinned 4 bean mix	Edgell		420g			
Tinned diced/chopped tomatoes, in tomato juice	Ardmona		400g			
Fruit salad, canned in juice	Goulburn Valley		700g			
Tinned steak & vegetables	Harvest		425g			
Tinned baked beans, in tomato sauce	Heinz		420g			
Tinned chicken & vegetable soup, ready to eat	Campbell's Country Ladle		505g			
Tuna, canned in vegetable oil, unflavoured (cheapest specified brand)	John West, Greenseas or Sirena		185g			
Pantry Foods						
Wholemeal Bread	Tip Top Sunblest		650g			
White Bread	Tip Top Sunblest		650g			
Muffin, commercial, uniced	Supermarket		$/100g			
Rolled oats, whole, Traditional (not quick oats)	Uncle Toby's		1kg			
Cornflakes	Kellogg's		725g			
Weetbix	Sanitarium		375g			
Spaghetti (white)	San Remo		500g			
White rice, medium grain	SunRice		1kg			
2 Minute noodles, chicken (cheapest specified brand)	Maggi or Fantastic		70g			
White Sugar	CSR		2kg			

Store name ___________________ Store Location: ________________ Date: ___________ Collector:_______

Food	Specific brand	Your brand	Specific size	Your size	Your cost	Comments
Cream-filled biscuit	Arnott's Monte-Carlo		250g			
Chewy Choc Chip Muesli Bar	Uncle Toby's		6x30g (185g)			
Water Crackers, plain	Arnott's		125g			
Savoury flavoured biscuits	Arnott's BBQ Shapes		175g			
Peanuts – roasted, unsalted peanuts	Cheapest branded		250g			
Mixed nuts, (incl. peanut), salted	Nobby's		375g			
Mint confectionary	Allen's Minties		150g			
Dairy milk chocolate, block	Cadbury		200g			
Chips/crisps, original, salted	Smith's or Thins		170g			
French Dressing, regular fat	Praise		330mL			
Tomato sauce, regular (not ketchup)	Heinz Big Red or Masterfoods		500mL			
Sunflower oil	Crisco		750mL			
Olive oil, Traditional (not extra virgin)	Moro		1 Litre			
Meats						
Lean beef mince (not heart smart)	Pre-pack(not vacuum)		per kg			
Lamb loin chops	Pre-pack		per kg			
Beef rump steak	Pre-pack		per kg			
Beef Sausages, pre-pack	Supermarket		per kg			
Refrigerated Items						
Cheddar cheese, regular fat	Coon		250g			
Cheddar cheese, reduced fat	Coon		250g			
Butter, original, salted (foil pack)	Western Star		250g			
Canola Margarine, regular fat	MeadowLea		500g			
Full cream milk, fresh	Paul's or Dairy Farmers		2L			
Reduced fat milk, fresh (not skim)	Paul's Trim or Dairy Farmers Lite		2L			
Chocolate Milk, regular fat	Breaka, Big M,Oak or Paul's		600mL			
Plain Yoghurt, natural, Greek, regular fat (~4% fat)	Jalna		1kg			
Yoghurt, vanilla/flavoured, reduced fat (~1% fat)	Jalna		1kg			
Leg Ham, pre-pack	Don's		250g			
Eggs, dozen, Free Range	Sunnyqueen Farms		700g			

Food	Specific brand	Your brand	Specific size	Your size	Your cost	Comments
Drinks						
Bottled water, still	Mt Franklin		600mL			
Soft drink, Cola	Coca Cola		1.25L			
Diet soft drink, Cola	Coca Cola		1.25L			
Orange Juice, Australian Grown (Fresh, chilled)	Berri		2L			
Frozen Foods						
Frozen mixed vegetables (cheapest specified brand)	Heinz, Birdseye or McCain		500g			
Frozen peas (cheapest specified brand)	Edgell, Birdseye or McCain		500g			
Beef lasagne, frozen	McCain		400g			
White crumbed fish fillet, frozen	Birds Eye		425g			
Vanilla Ice cream, regular fat	Nestle Peters Original		2L			
Other Items						
Whole Barbeque Chicken, cooked - Large/ Family	Supermarket		Per unit ~1.5kg			
Pre-made Chicken & Salad Sandwich (wholemeal) (1 sandwich = ~220g)	Supermarket or, if unavailable, at closest garage/service station		2sl bread + filling (triangle pre-pack)			

Items from other stores:

Food	Store	Your store	Specific size	Your size	Your cost	Comments
Cooked hot potato chips	Independent Fish & Chip shop		~110g 1 serve			
Beef hamburger (Big Mac)	McDonald's		1 burger			
Beef Pie, single serve, full pastry	Independent Bakery		~250g 1 pie			
Supreme Pizza, thin base (1 pizza=0.55kg)	Pizza Hut		1 large pizza			

Liquor Store Name: ______________________

Food	Specific brand	Your brand	Specific size	Your size	Your cost	Comments
Beer	VB		6 x 375mL			
Sparkling white wine	Yellow		750mL			
Whisky	Johnny Walker Red Label		700mL			
Red wine	Penfolds Koonungara Hill Shiraz		750mL			

Supplementary File S4. Total diet and component costs for a reference household per fortnight in each included location in Greater Brisbane

Table S4A: Total diet and component costs for a reference household per fortnight in each included location in Greater Brisbane, SEIFA quintile 1

**Location**	**Comm1**						**Comm2**						**Comm3**					
**Area of socioeconomic disadvantage**	Most disadvantaged area (SEIFA quintile 1)						Most disadvantaged area (SEIFA quintile 1)						Most disadvantaged area (SEIFA quintile 1)					
	**Current diet**			**Recommended diet**			**Current diet**			**Recommended diet**			**Current diet**			**Recommended diet**		
**Year**	**2019**	**2020**	**Change in cost (A$)**	**2019**	**2020**	**Change in cost (A$)**	**2019**	**2020**	**Change in cost (A$)**	**2019**	**2020**	**Change in cost (A$)**	**2019**	**2020**	**Change in cost (A$)**	**2019**	**2020**	**Change in cost (A$)**
Food/food groups	**Mean cost (A$)**	**Mean cost (A$)**		**Mean cost (A$)**	**Mean cost (A$)**		**Mean cost (A$)**	**Mean cost (A$)**		**Mean cost (A$)**	**Mean cost (A$)**		**Mean cost (A$)**	**Mean cost (A$)**		**Mean cost (A$)**	**Mean cost (A$)**	
Water, bottled	22.80	21.89	-0.91	22.80	21.89	-0.91	22.95	19.80	-3.15	22.95	19.80	-3.15	17.33	20.01	+2.68	17.33	20.01	+2.68
Fruit	54.53	57.54	+3.01	68.81	83.62	+14.81	49.75	52.94	+3.19	60.67	76.83	+16.16	56.23	59.65	+3.42	83.23	90.80	+7.57
Vegetables (& legumes)	42.34	41.66	-0.68	109.35	100.15	-9.20	43.08	40.94	-2.14	107.99	98.61	-9.38	44.43	41.19	-3.24	114.37	99.13	-15.24
Grain (cereal) foods	41.66	47.07	+5.41	109.60	116.78	+7.18	45.39	44.65	-0.73	112.32	109.98	-2.34	42.83	45.07	+2.24	108.29	111.59	+3.30
Lean meats, poultry, fish, eggs, nuts, seeds & alternatives	98.23	101.24	+3.01	187.94	191.53	+3.59	99.19	100.58	+1.38	188.22	197.91	+9.69	91.74	99.21	+7.47	176.15	188.41	+12.26
Milk, yoghurt, cheese & alternatives	49.40	54.44	+5.04	110.85	122.39	+11.54	48.40	55.19	+6.80	111.00	117.73	+6.74	47.81	55.77	+7.96	110.34	123.84	+13.50
Unsaturated oils and spreads	1.31	1.31	0.00	8.44	8.41	-0.03	1.25	1.26	+0.02	8.07	8.61	+0.54	1.25	1.27	+0.03	8.08	9.09	+1.00
Artificially sweetened beverages	5.80	6.56	+0.76	-	-	-	4.86	6.02	+1.16	-	-	-	6.23	6.02	-0.20	-	-	-
Sugar sweetened beverages	32.64	32.64	0.00	-	-	-	29.31	30.27	+0.96	-	-	-	32.96	30.27	-2.69	-	-	-
Takeaway foods	140.76	149.72	+8.96	-	-	-	143.31	151.25	+7.94	-	-	-	145.24	150.84	+5.61	-	-	-
Alcoholic beverages	81.71	89.42	+7.70	-	-	-	99.79	99.15	-0.63	-	-	-	99.79	99.15	-0.63	-	-	-
All other discretionary choices	191.01	188.35	-2.66	-	-	-	181.56	178.26	-3.30	-	-	-	184.90	187.27	+2.37	-	-	-
**Total diet**	**762.20**	**791.84**	**+29.64**	**617.79**	**644.77**	**+26.97**	**768.82**	**780.32**	**+11.50**	**611.21**	**629.47**	**+18.26**	**770.73**	**795.73**	**+25.00**	**617.79**	**642.85**	**+25.07**
Healthy foods and drinks	316.07	331.71	+15.64	617.79	644.77	+26.97	314.85	321.38	+6.53	611.21	629.47	+18.26	307.85	328.19	+20.35	617.79	642.85	+25.07
Discretionary foods and drinks	446.13	460.13	+14.00	-	-	-	453.96	458.94	+4.97	-	-	-	462.88	467.54	+4.65	-	-	-

Table S4B: Total diet and component costs for a reference household per fortnight in each included location in Greater Brisbane, SEIFA quintile 3

**Location**	**Comm4**						**Comm5**						**Comm6**						**Comm7**					
**Area of socio-economic disadvantage**	Median disadvantaged area (SEIFA quintile 3)						Median disadvantaged area (SEIFA quintile 3)						Median disadvantaged area (SEIFA quintile 3)						Median disadvantaged area (SEIFA quintile 3)					
	**Current diet**			**Recommended diet**			**Current diet**			**Recommended diet**			**Current diet**			**Recommended diet**			**Current diet**			**Recommended diet**		
**Year**	**2019**	**2020**	**Change in cost (A$)**	**2019**	**2020**	**Change in cost (A$)**	**2019**	**2020**	**Change in cost (A$)**	**2019**	**2020**	**Change in cost (A$)**	**2019**	**2020**	**Change in cost (A$)**	**2019**	**2020**	**Change in cost (A$)**	**2019**	**2020**	**Change in cost (A$)**	**2019**	**2020**	**Change in cost (A$)**
Food/food groups	**Mean cost (A$)**	**Mean cost (A$)**		**Mean cost (A$)**	**Mean cost (A$)**		**Mean cost (A$)**	**Mean cost (A$)**		**Mean cost (A$)**	**Mean cost (A$)**		**Mean cost (A$)**	**Mean cost (A$)**		**Mean cost (A$)**	**Mean cost (A$)**		**Mean cost (A$)**	**Mean cost (A$)**		**Mean cost (A$)**	**Mean cost (A$)**	
Water, bottled	20.83	19.86	-0.97	20.83	19.86	-0.97	20.15	18.39	-1.76	20.15	18.39	-1.76	21.19	19.62	-1.57	21.19	19.62	-1.57	18.98	16.89	-2.09	18.98	16.89	-2.09
Fruit	50.68	55.47	+4.79	76.88	84.65	+7.77	58.09	56.69	-1.40	82.85	86.42	3.57	51.97	56.47	+4.49	73.66	88.93	+15.27	50.19	57.61	+7.43	67.52	90.29	+22.77
Vegetables (& legumes)	40.63	40.89	+0.26	102.39	96.37	-6.02	45.52	40.18	-5.34	115.21	94.45	-20.75	41.48	37.13	-4.36	104.45	86.88	-17.57	44.66	40.64	-4.02	113.42	97.62	-15.80
Grain (cereal) foods	42.44	45.48	+3.04	107.24	111.12	+3.88	43.50	43.71	+0.22	109.68	110.15	+0.47	43.77	46.04	+2.27	108.64	112.42	+3.78	44.26	46.25	+2.00	110.55	116.31	+5.76
Lean meats, poultry, fish, eggs, nuts, seeds & alternatives	99.19	99.63	+0.44	192.30	195.88	+3.58	99.78	97.54	-2.24	193.55	190.23	-3.33	91.58	94.90	+3.32	172.28	181.29	+9.02	96.76	104.38	+7.62	181.94	199.65	+17.70
Milk, yoghurt, cheese & alternatives	48.28	51.40	+3.12	110.88	107.52	-3.36	49.63	56.12	+6.49	113.72	120.87	+7.15	41.29	55.06	+13.77	99.56	120.53	+20.97	46.03	55.57	+9.55	107.10	124.34	+17.24
Unsaturated oils and spreads	1.23	1.27	+0.05	8.55	9.05	+0.49	1.26	1.27	0.00	8.81	8.83	+0.02	1.26	1.24	-0.01	8.57	7.80	-0.77	1.19	1.26	+0.07	7.89	8.58	+0.69
Artificially sweetened soft drink	6.02	6.02	0.00	-	-	-	5.59	6.02	+0.43	-	-	-	6.01	6.02	+0.02	-	-	-	4.94	6.02	+1.08	-	-	-
Sugar sweetened beverages	29.79	30.27	+0.48	-	-	-	29.79	30.27	+0.48	-	-	-	33.54	30.27	-3.27	-	-	-	29.79	30.27	+0.48	-	-	-
Takeaway foods	154.39	167.80	+13.41	-	-	-	146.51	151.63	+5.12	-	-	-	164.25	171.17	+6.92	-	-	-	145.36	156.09	+10.73	-	-	-
Alcoholic beverages	99.79	99.15	-0.63	-	-	-	99.79	99.15	-0.63	-	-	-	95.30	98.12	+2.82	-	-	-	99.79	99.15	-0.63	-	-	-
All other discretionary choices	170.57	194.82	+24.25	-	-	-	193.48	184.98	-8.50	-	-	-	173.48	172.73	-0.75	-	-	-	174.62	176.57	+1.94	-	-	-
**Total diet**	**763.83**	**812.07**	**+48.24**	**619.08**	**624.44**	**+5.37**	**793.08**	**785.95**	**-7.13**	**643.97**	**629.34**	**-14.64**	**765.11**	**788.76**	**+23.66**	**588.34**	**617.48**	**+29.14**	**756.56**	**790.71**	**+34.15**	**607.39**	**653.67**	**+46.27**
Healthy foods and drinks	309.30	320.02	+10.72	619.08	624.44	+5.37	323.52	319.92	-3.60			-14.64	298.55	316.48	+17.93	588.34	617.48	+29.14	307.00	328.63	+21.64	607.39	653.67	+46.27
Discretionary foods and drinks	454.53	492.04	+37.51	-	-	-	469.57	466.03	-3.53	-	-	-	466.56	472.29	+5.73	-	-	-	449.56	462.08	+12.52	-	-	-

Table S4C: Total diet and component costs for a reference household per fortnight in each included location in Greater Brisbane, SEIFA quintile 5

**Location**	**Comm8**						**Comm9**						**Comm10**					
**Area socioeconomic disadvantage**	Least disadvantaged area (SEIFA quintile 5)						Least disadvantaged area (SEIFA quintile 5)						Least disadvantaged area (SEIFA quintile 5)					
	**Current diet**			**Recommended diet**			**Current diet**			**Recommended diet**			**Current diet**			**Recommended diet**		
**Year**	**2019**	**2020**		**2019**	**2020**		**2019**	**2020**		**2019**	**2020**		**2019**	**2020**		**2019**	**2020**	
**Food/food groups**	**Mean cost (A$)**	**Mean cost (A$)**	**Change in cost (A$)**	**Mean cost (A$)**	**Mean cost (A$)**	**Change in cost (A$)**	**Mean cost (A$)**	**Mean cost (A$)**	**Change in cost (A$)**	**Mean cost (A$)**	**Mean cost (A$)**	**Change in cost (A$)**	**Mean cost (A$)**	**Mean cost (A$)**	**Change in cost (A$)**	**Mean cost (A$)**	**Mean cost (A$)**	**Change in cost (A$)**
Water, bottled	20.30	15.42	-4.88	20.30	15.42	-4.88	18.98	20.12	+1.14	18.98	20.12	+1.14	20.01	18.36	-1.65	20.01	18.36	-1.65
Fruit	49.80	57.78	+7.98	67.60	88.93	+21.33	62.32	58.38	-3.95	83.17	90.44	+7.27	50.28	61.87	+11.60	63.72	102.20	+38.49
Vegetables (& legumes)	42.94	38.58	-4.36	109.58	93.80	-15.78	47.35	40.12	-7.24	121.05	97.52	-23.53	43.51	43.81	+0.30	105.85	105.20	-0.65
Grain (cereal) foods	44.33	45.84	+1.51	109.36	111.40	+2.03	46.40	49.84	+3.44	108.36	123.31	+14.95	48.81	47.71	-1.10	115.84	115.49	-0.35
Lean meats, poultry, fish, eggs, nuts, seeds & alternatives	92.80	106.40	+13.60	179.36	205.14	+25.77	102.12	106.26	+4.15	199.54	201.82	+2.28	93.10	108.45	+15.35	173.89	206.47	+32.58
Milk, yoghurt, cheese & alternatives	48.88	55.53	+6.65	110.12	121.53	+11.41	52.52	56.33	+3.81	133.51	129.48	-4.03	47.10	54.76	+7.65	118.86	120.54	+1.69
Unsaturated oils and spreads	1.25	1.26	+0.01	8.23	8.65	+0.42	1.35	1.38	+0.03	9.22	8.02	-1.20	1.40	1.44	+0.04	8.33	8.82	+0.49
Artificially sweetened beverages	5.69	6.02	+0.33	-	-	-	5.89	6.56	+0.67	-	-	-	5.38	6.11	+0.73	-	-	-
Sugar sweetened beverages	30.27	30.27	0.00	-	-	-	32.96	32.96	0.00	-	-	-	30.40	30.72	+0.32	-	-	-
Takeaway foods	158.58	165.66	+7.09	-	-	-	147.47	154.85	+7.38	-	-	-	147.28	158.55	+11.27	-	-	-
Alcoholic beverages	99.79	98.74	-1.05	-	-	-	88.11	98.12	+10.01	-	-	-	99.79	99.15	-0.63	-	-	-
All other discretionary choices	170.81	176.36	+5.55	-	-	-	199.13	187.25	-11.88	-	-	-	184.62	187.23	+2.61	-	-	-
**Total diet**	**765.44**	**797.88**	**+32.44**	**604.55**	**644.85**	**+40.30**	**804.61**	**812.16**	**+7.55**	**673.82**	**670.72**	**-3.11**	**771.67**	**818.16**	**+46.49**	**606.48**	**677.09**	**+70.60**
Healthy foods and drinks	305.99	326.84	+20.85	604.55	644.85	+40.30	336.93	338.98	+2.04	673.82	670.72	-3.11	309.58	342.51	+32.93	606.48	677.09	+70.60
Discretionary foods and drinks	459.45	471.04	+11.59	-	-	-	467.67	473.18	+5.51	-	-	-	462.09	475.65	+13.56	-	-	-

Supplementary File S5. Total diet and component costs for a reference household per fortnight in Greater Brisbane by area of socioeconomic disadvantage

Table S5A: Total current diet and component costs for a reference household per fortnight in Greater Brisbane by area of socioeconomic disadvantage

Location	Most disadvantaged areas (SEIFA quintile 1), n=9 locations						Median disadvantaged areas (SEIFA quintile 3), n=12 locations						Least disadvantaged areas (SEIFA quintile 5), n=9 locations					
Year	2019		2020		Change in cost (A$)	Change in cost (%)	2019		2020		Change in cost (A$)	Change in cost (%)	2019		2020		Change in cost (A$)	Change in cost (%)
Food/food groups	Mean cost (A$) ±SD	Proportion of total cost (%)	Mean cost (A$) ± SD	Proportion of total cost (%)			Mean cost (A$) ± SD	Proportion of total cost (%)	Mean cost (A$) ± SD	Proportion of total cost (%)			Mean cost (A$) ± SD	Proportion of total cost (%)	Mean cost (A$) ± SD	Proportion of total cost (%)		
Water, bottled	$21.03 ±2.62	2.74%	$20.57 ±0.94	2.61%	-0.46	-2.19%	$20.29 ±0.84	2.64%	$18.69 ±1.18	2.35%	-1.60	-7.88%	$19.76 ±0.57	2.53%	$17.97 ±1.94	2.22%	-1.80	-9.09%
Fruit	$53.50 ±2.75	6.97%	$56.71 ±2.8	7.18%	+3.21	+5.99%	$52.73 ±3.16	6.85%	$56.56 ±0.76	7.12%	+3.83	+7.26%	$54.13 ±5.79	6.94%	$59.34 ±1.80	7.33%	+5.21	+9.62%
Vegetables (& legumes)	$43.28 ±0.87	5.64%	$41.26 ±0.3	5.23%	-2.02	-4.67%	$43.07 ±2.06	5.60%	$39.71 ±1.51	5.00%	-3.36	-7.81%	$44.6 ±1.96	5.71%	$40.84 ±2.19	5.05%	-3.76	-8.44%
Grain (cereal) foods	$43.29 ±1.56	5.64%	$45.60 ±1.06	5.78%	+2.30	+5.32%	$43.49 ±0.67	5.65%	$45.37 ±1.00	5.71%	+1.88	+4.33%	$46.51 ±1.83	5.96%	$47.79 ±1.63	5.90%	+1.28	+2.76%
Lean meats, poultry, fish, eggs, nuts, seeds & alternatives	$96.39 ±3.31	12.56%	$100.34 ±0.84	12.71%	+3.95	+4.10%	$96.82 ±3.24	12.58%	$99.11 ±3.47	12.48%	+2.28	+2.36%	$96.01 ±4.32	12.30%	$107.04 ±1.00	13.22%	+11.03	+11.49%
Milk, yoghurt, cheese & alternatives	$48.53 ±0.65	6.33%	$55.13 ±0.54	6.99%	+6.60	+13.60%	$46.31 ±3.17	6.02%	$54.54 ±1.85	6.87%	+8.23	+17.78%	$49.50 ±2.26	6.34%	$55.54 ±0.64	6.86%	+6.04	+12.19%
Unsaturated oils and spreads	$1.27 ±0.03	0.17%	$1.28 ±0.02	0.16%	+0.02	+1.23%	$1.23 ±0.03	0.16%	$1.26 ±0.01	0.16%	+0.03	+2.24%	$1.33 ±0.06	0.17%	$1.36 ±0.07	0.17%	+0.03	+1.97%
Artificially sweetened beverages	$5.63 ±0.57	0.73%	$6.20 ±0.25	0.79%	+0.57	+10.17%	$5.64 ±0.44	0.73%	$6.02 ±0.00	0.76%	+0.38	+6.77%	$5.65 ±0.21	0.72%	$6.23 ±0.23	0.77%	+0.58	+10.22%
Sugar sweetened beverages	$31.64 ±1.65	4.12%	$31.06 ±1.12	3.94%	-0.58	-1.82%	$30.73 ±1.62	3.99%	$30.27 ±0.00	3.81%	-0.46	-1.48%	$31.21 ±1.24	4.00%	$31.32 ±1.18	3.87%	+0.11	+0.34%
Takeaway foods	$143.10 ±1.83	18.65%	$150.61 ±0.65	19.08%	+7.50	+5.24%	$152.63 ±7.56	19.83%	$161.67 ±8.06	20.35%	+9.05	+5.93%	$151.11 ±5.28	19.36%	$159.69 ±4.49	19.73%	+8.58	+5.68%
Alcoholic beverages	$93.76 ±8.52	12.22%	$95.91 ±4.59	12.15%	+2.15	+2.29%	$98.66 ±1.94	12.82%	$98.89 ±0.45	12.45%	+0.23	+0.23%	$95.90 ±5.5	12.29%	$98.67 ±0.43	12.19%	+2.78	+2.89%
All other discretionary choices	$185.82 ±1.65	24.22%	$184.63 ±4.53	23.39%	-1.20	-0.64%	$178.04 ±1.62	23.13%	$182.27 ±8.49	22.95%	+4.24	+2.38%	$184.86 ±1.24	23.68%	$183.62 ±5.13	22.69%	-1.24	-0.67%
**Total diet**	**$767.25 ±3.66**	**100.00%**	**$789.30 ±6.55**	**100.00%**	**+22.05**	**+2.87%**	**$769.64 ±13.92**	**100.00%**	**$794.37 ±10.35**	**100.00%**	**+24.73**	**+3.21%**	**$780.57 ±17.19**	**100.00%**	**$809.40 ±8.51**	**100.00%**	**+28.83**	**+3.69%**
Healthy foods and drinks	$312.92 ±3.62	40.79%	$327.09 ±4.29	41.44%	+14.17	+4.53%	$309.59 ±8.98	40.22%	$321.26 ±4.49	40.44%	+11.67	+3.77%	$317.5 ±13.82	40.68%	$336.11 ±6.71	41.53%	+18.61	+5.86%
Discretionary foods and drinks	$454.33 ±6.85	59.21%	$462.20 ±3.8	58.56%	+7.88	+1.73%	$460.06 ±8.27	59.78%	$473.11 ±11.52	59.56%	+13.06	+2.84%	$463.07 ±3.43	59.32%	$473.29 ±1.88	58.47%	+10.22	+2.21%

Table S5B: Total recommended diet and component costs for a reference household per fortnight in Greater Brisbane by area of socioeconomic disadvantage

**Location**	**Most disadvantaged areas (SEIFA quintile 1), n=9 locations**						**Median disadvantaged areas (SEIFA quintile 3), n=12 locations**						**Least disadvantaged areas (SEIFA quintile 5), n=9 locations**					
**Year**	**2019**		**2020**		**Change in cost (A$)**	**Change in cost (%)**	**2019**		**2020**		**Change in cost (A$)**	**Change in cost (%)**	**2019**		**2020**		**Change in cost (A$)**	**Change in cost (%)**
**Food/ food groups**	**Mean cost (A$) ±SD**	**Proportion of total cost (%)**	**Mean cost (A$) ±SD**	**Proportion of total cost (%)**			**Mean cost (A$) ±SD**	**Proportion of total cost (%)**	**Mean cost (A$) ±SD**	**Proportion of total cost (%)**			**Mean cost (A$) ±SD**	**Proportion of total cost (%)**	**Mean cost (A$) ±SD**	**Proportion of total cost (%)**		
Water, bottled	$21.03 ±2.62	3.42%	$20.57 ±0.94	3.22%	-0.46	-2.19%	$20.29 ±0.84	3.30%	$18.69 ±1.18	2.96%	-1.60	-7.88%	$19.76 ±0.57	3.15%	$17.97 ±1.94	2.70%	-1.80	-9.09%
Fruit	$70.90 ±9.33	11.52%	$83.75 ±5.70	13.11%	+12.85	+18.12%	$75.23 ±5.54	12.24%	$87.57 ±2.19	13.87%	+12.35	+16.41%	$71.49 ±8.41	11.38%	$93.86 ±5.94	14.13%	+22.36	+31.28%
Vegetables (& legumes)	$110.57 ±2.74	17.96%	$99.29 ±0.64	15.54%	-11.27	-10.20%	$108.87 ±5.53	17.71%	$93.83 ±4.17	14.86%	-15.04	-13.81%	$112.16 ±6.47	17.85%	$98.84 ±4.75	14.88%	-13.32	-11.87%
Grain (cereal) foods	$110.07 ±1.68	17.88%	$112.78 ±2.90	17.65%	+2.71	+2.47%	$109.03 ±1.24	17.74%	$112.50 ±2.35	17.82%	+3.47	+3.18%	$111.19 ±3.31	17.70%	$116.74 ±4.94	17.57%	+5.55	+4.99%
Lean meats, poultry, fish, eggs, nuts, seeds & alternatives	$184.10 ±5.63	29.91%	$192.62 ±3.95	30.14%	+8.51	+4.62%	$185.02 ±8.63	30.10%	$191.76 ±6.91	30.38%	+6.74	+3.64%	$184.26 ±11.03	29.33%	$204.47 ±1.96	30.78%	+20.21	+10.97%
Milk, yoghurt, cheese & alternatives	$110.73 ±0.28	17.99%	$121.32 ±2.61	18.99%	+10.59	+9.57%	$107.82 ±5.31	17.54%	$118.31 ±6.41	18.74%	+10.50	+9.74%	$120.83 ±9.65	19.23%	$123.85 ±4.00	18.65%	+3.02	+2.50%
Unsaturated oils and spreads	$8.20 ±0.17	1.33%	$8.70 ±0.28	1.36%	+0.50	+6.14%	$8.46 ±0.35	1.38%	$8.57 ±0.47	1.36%	+0.11	+1.31%	$8.59 ±0.45	1.37%	$8.50 ±0.34	1.28%	-0.10	-1.12%
**Total diet**	**$615.60 ±3.10**	**100.00%**	**$639.03 ±6.80**	**100.00%**	**+23.43**	**+3.81%**	**$614.70 ±20.15**	**100.00%**	**$631.23 ±13.62**	**100.00%**	**+16.54**	**+2.69%**	**$628.29 ±32.21**	**100.00%**	**$664.22 ±13.94**	**100.00%**	**+35.93**	**+5.72%**

Supplementary File S6. Differences between the mean total cost of the current and recommended diets by area socioeconomic disadvantage and within area of socioeconomic disadvantage, t-tests

Table S6A: The differences between the mean total cost of the current and recommended diets by area of socioeconomic disadvantage, p-values of the t-tests

	2019		2020	
**Area of socioeconomic disadvantage**	**Current diet**	**Recommended diet**	**Current diet**	**Recommended diet**
Most vs median disadvantaged (SEIFA quintile 1 vs quintile 3)	0.85	0.93	0.69	0.44
Most vs least disadvantaged (SEIFA quintile 1 vs quintile 5)	0.55	0.60	0.32	0.23
Median vs least disadvantaged (SEIFA quintile 3 vs quintile 5)	0.62	0.59	0.41	0.13

Table S6B: The differences between the mean total cost of the current and recommended diets within area of socioeconomic disadvantage between 2019 and 2020, p-values of the t-tests (* indicates p<0.05, ** indicates p<0.01)

	2019 vs 2020	
**Area of socioeconomic disadvantage**	**Current diet**	**Recommended diet**
Most disadvantaged (SEIFA quintile 1, n=9)	0.15	<0.01**
Median disadvantaged (SEIFA quintile 3, n=12)	<0.03*	0.15
Least disadvantaged (SEIFA quintile 5, n=9)	0.28	0.24

Supplementary File S7. Affordability of current and recommended diets for a reference household with minimum wage and welfare-only disposable household income in Greater Brisbane by area of socioeconomic disadvantage

Table S7A: Affordability of current and recommended diets for a reference household with minimum wage disposable household income in Greater Brisbane by area of socioeconomic disadvantage

**Area of socioeconomic disadvantage**	**Affordability of current diet** (% of income)				**Affordability of recommended diet** (% of income)			
	**2019**	**2020**	**Absolute change**	**Relative change**	**2019**	**2020**	**Absolute change**	**Relative change**
Most disadvantaged areas (SEIFA quintile 1)	33%	24%	-9%	-27%	26%	19%	-7%	-27%
Median disadvantaged areas (SEIFA quintile 3)	33%	24%	-9%	-27%	26%	19%	-7%	-27%
Least disadvantaged areas (SEIFA quintile 5)	33%	24%	-9%	-27%	27%	20%	-7%	-25%

Table S7B: Affordability of current and recommended diets for a reference household with welfare-only disposable household income in Greater Brisbane by area of socioeconomic disadvantage

**Area of socioeconomic disadvantage**	**Affordability of current diet** (% of income)				**Affordability of recommended diet** (% of income)			
	**2019**	**2020**	**Absolute change**	**Relative change**	**2019**	**2020**	**Absolute change**	**Relative change**
Most disadvantaged areas (SEIFA quintile 1)	44%	26%	-19%	-42%	35%	21%	-15%	-41%
Median disadvantaged areas (SEIFA quintile 3)	44%	26%	-18%	-42%	35%	20%	-15%	-42%
Least disadvantaged areas (SEIFA quintile 5)	45%	26%	-19%	-42%	36%	22%	-15%	-40%
